# Klotho and Renal Fibrosis

**DOI:** 10.5812/numonthly.16179

**Published:** 2013-11-13

**Authors:** Sepide Zununi Vahed, Parisa Nikasa, Mohammadreza Ardalan

**Affiliations:** 1 Faculty of Advanced Medical Sciences, Tabriz University of Medical Sciences, Tabriz, IR Iran; 2 Chronic Kidney Disease Research Center, Tabriz University of Medical Sciences, Tabriz, IR Iran

**Keywords:** Klotho Protein, Nephrogenic Fibrosing Dermopathy, Renal Insufficiency, Chronic, Fibrosis

Renal fibrosis is a pathological condition that characterized by excessive accumulation of extracellular matrix (ECM) and it is a common pathway of progression in different renal diseases including chronic glomerulonephritis, diabetic nephropathy chronic allograft nephropathy and renal senescence (1, 2). In the process of renal fibrosis leukocyte infiltration, activation of fibroblasts and myofibroblasts, epithelial-mesenchymal transition (EMT) all have been identified as the major events (3). Behind The pathological features there are complex orchestrated interplay of different factors including cytokines (TGF-β, IFN-γ, IL-10), transcriptional activator factors (Sp1, Egr-1, Smad3), cell surface repressors down regulation or up regulation (Smad7, Fli-1, PPAR-γ, p53, Klotho) and epigenetic modulators (acetyltransferase, methyltransferases, deacetylases, microRNAs) (4). Deregulation of pro-fibrotic and down regulation of anti-fibrotic factors play an important role in initiation and progression of renal fibrosis. Understanding the mechanism would be valuable to diagnose, prevent and even reverse the process of fibrosis. The role of micro RNA as a biomarker or as a therapeutic modality of renal fibrosis has recently be discussed by authors (5).

Klotho, was first identified as an anti-aging protein in 1997, and it spawned two decades of aging research (6). Klotho is expressed in different tissues including kidney, heart, brain, and parathyroid but it is particularly highly expressed in kidney and predominantly in distal and proximal convoluted tubules. Klotho is a single-pass transmembrane (130 KDa) protein (6, 7). Membrane-anchored Klotho acts as a co-receptor for fibroblast growth factor-23 that promote renal phosphate excretion and suppresses active vitamin D synthesis (8, 9) soluble Klotho (70 kD) is generated from the *Klotho* gene through alternative splicing. It could also sheds from cell membrane (9). Soluble Klotho (sKl) acts as a hormonal with multiple renal and extrarenal functions including suppression of fibrosis (10), antioxidant activity (11), preservation of stem cells (12), down regulation of Wnt signaling and acting as an anti-aging agent (13) and modulation of ion transport (14). Klotho-deficient mice develop severe kidney damage and fibrosis. Kl^−/−^ deficient mice with unilateral ureteral obstruction (UUO) had higher TGF-β1 levels and more renal fibrosis than WT wild type mice with UUO (13, 15) in human kidney, decreased Klotho expression occurs in early stages of CKD, and could be responsible for early increase in serum FGF23 level (16). Klotho deficient animals develops accelerated vascular calcification (17). Cell culture studies have confirmed that Klotho deficiency directly promotes renal epithelial cell senescence (14, 18, 19).

Experimental studies have revealed that Klotho level decreases in acute and chronic kidney diseases (12) and Klotho has a nephron-protective role (6, 9, 10, 12, 20). Klotho acts as an endogenous inhibitor of multiple growth factor including transforming growth factor-β1 (TGF-ß1), Wnt, and IGF-1 (8, 10, 12). Klotho primarily suppresses renal fibrosis through inhibition of TGF-ß1 signaling (21), and plays a central role in the pathogenesis of renal fibrosis in both experimental and human kidney diseases (22, 23). TGF-β1 induces matrix production and inhibits ECM degradation via suppression of matrix metalloproteinases (MMPs) and induction of the of MMPs inhibitors (TIMPs) (4, 16). TGF-β1 inhibits expression of genes that are essential for epithelial phenotype such as E-cadherin, and induces expression of factors that are essential for mesenchymal phenotype such as vimentin, collagen-1, and N-cadherin respectively (17). Secreted Klotho hinders profibrotic effects of TGF-β1 through direct interaction with TGF-β receptor II (8). TGF-β1 itself could suppress the tissue expression of Klotho (10, 12, 18). Klotho is a critical negative regulator of Wnt signaling that not only acting as an anti-aging agent (13), but also it has a critical role in normal wound healing, and its sustained activation is associated with fibrogenesis (19, 24). Klotho binds to Wnt ligands and represses Wnt-induced transcription of β-catenin targets in response to TGF-β1. Invivo expression of secreted Klotho in inhibits the activation of renal β-catenin, and decreases ECM deposition (12). These results suggest that Klotho is an endogenous antagonist of Wnt and β-catenin activity that inhibits the activation of renal myofibroblast, EMT and renal fibrosis (12). Senescent cells secrete altered levels of growth factors that future increase susceptibility to apoptosis, and delay the repair and regeneration in the aging kidney (5, 23). From histological point of view, renal senescence is characterized by reduction in cortical mass, glomerulosclerosis, interstitial fibrosis/tubular atrophy (IF/TA) and arteriosclerosis (21, 25). Telomere shortening and cell cycle arrest are physiologic contributors of aging and from pathophysiologic causes; oxidative stress, epigenetic alterations, mitochondrial injury and immunosuppressive agent are important contributors (2, 25, 26). Soluble klotho binds to Wnt protein and inhibits cell senescence through the inhibition of Wnt signaling (13). Maekawa et al. showed the anti-senescence properties of exogenous administrated Klotho (27). Therefore, it could be considered a therapeutic modality for prevention of IF/TA in renal allograft.

Increased activity of plasminogen activator inhibitor 1 (PAI-1) is associated with kidney fibrosis it can be seen in chronic allograft nephropathy, chronic glomerulonephritis, diabetic nephropathy, and hypertensive nephrosclerosis (28, 29). PAI-1 mRNA and PAI-1 protein activity were strikingly elevated in multiple tissues of Klotho-deficient (Kl^−/−^) mice (9), and Klotho supplementation might reduce PAI-1 activity (9). Sugiura et al. (2010) reported that Kl^−/−^ deficient mice had higher levels of renal tubulointerstitial fibrosis that was associated with upregulation of TGF-β1 (30).

Klotho supplementation inhibits renal fibrosis by suppression of fibrotic markers includings; α-smooth muscle actin, Fibronectin, Vimentin, collagen-1, MMPs. and TGF-β1 (8). Soluble Klotho can suppress renal fibrosis and preserve renal function in UUO model of renal injury, therefore it could be considered as a novel therapeutic agent in renal fibrosis (8). Chronic kidney disease (CKD) and aging going hand in hand. Indoxyl sulfate is a uremic toxin that reduces renal Klotho expression, and contributes to cell senescence and renal fibrosis (30, 31).

Increased methylation of Klotho promoter, microRNA-339 and microRNA-556 could directly decrease Klotho expression in cultured cells (32). Klotho promotes kidney regeneration after ischemia–reperfusion injury via the suppression of fibrosis-promoting growth factors, preservation of stem cells, and the recovery of endothelial integrity and function, (33) Klotho incubation mitigates cell senescence and apoptosis in endothelial cells triggered by oxidative stress or by Klotho deficiency (10, 19). These properties could be considered as therapeutic measurements in future.

Klotho opens a new venue in renal fibrosis research. it is a promising diagnostic marker, a prognostic marker and a therapeutic agent (9, 20). Preventing its down regulation by targeting the factors that decrease its expression, upregulation of its endogenous production or its external repletion would be applicable to develop new therapeutic strategies for renal fibrosis.
